# *Notes from the Field*: Acute Intoxications from Consumption of *Amanita muscaria* Mushrooms — Minnesota, 2018

**DOI:** 10.15585/mmwr.mm6821a4

**Published:** 2019-05-31

**Authors:** Joanne Taylor, Stacy Holzbauer, Danushka Wanduragala, Alexander Ivaskovic, Ron Spinosa, Kirk Smith, Justin Corcoran, Ashley Jensen

**Affiliations:** ^1^Epidemic Intelligence Service, CDC; ^2^Infectious Diseases, Epidemiology, Prevention and Control Division, Minnesota Department of Health; ^3^Career Epidemiology Field Office Program, Office of Public Health Preparedness and Response, CDC; ^4^HealthEast Care System, Saint Paul, Minnesota; ^5^Minnesota Mycological Society, Eagan, Minnesota; ^6^Minnesota Poison Control System.

In October, 2018, a middle-aged ethnic Karen* man from Burma was evaluated at a hospital emergency department with altered mental status, vomiting, diarrhea, incontinence, sweating, swelling of the lip and tongue, and excessive salivation. Signs and symptoms began approximately 2–3 hours after eating mushrooms at home. The patient was admitted for supportive treatment, including endotracheal intubation for airway protection and mechanical ventilation because of acute respiratory failure and hypoxia for 4 days. His daughter, who also ate the mushrooms, was evaluated for similar, but milder symptoms, including mild sweating and nausea; she was admitted overnight for observation, and was discharged the following day. The father recovered and was discharged 8 days after admission.

An emergency department physician suspected possible cholinergic mushroom toxicosis and notified the Minnesota Poison Control System (MPCS). MPCS notified the Minnesota Department of Health (MDH), and, together with the Minnesota Mycological Society (MMS), they initiated an investigation. The patient’s medical records and exposure history were obtained and reviewed, and he was interviewed through a Karen language interpreter. He described the mushroom and collection location and later sent a photo of a mushroom that he said looked similar to the one he picked. He reported that he had picked mushrooms from an area near his workplace and cooked them in turmeric, oil, and water; only he and his daughter had eaten the mushrooms, and she reportedly consumed substantially fewer mushrooms than he did. He said this was the first time he had picked mushrooms since arriving in Minnesota in 2015 and that he had selected the mushrooms because they resembled the Ochre mushroom (*Amanita hemibapha* var. *ochracea)* from his native Burma. A site visit to the location where the mushrooms were picked identified one remaining mushroom that matched the patient’s description (Figure); an MMS mycologist examined the mushroom and identified it as *Amanita muscaria* var. *guessowii*.

**FIGURE:**
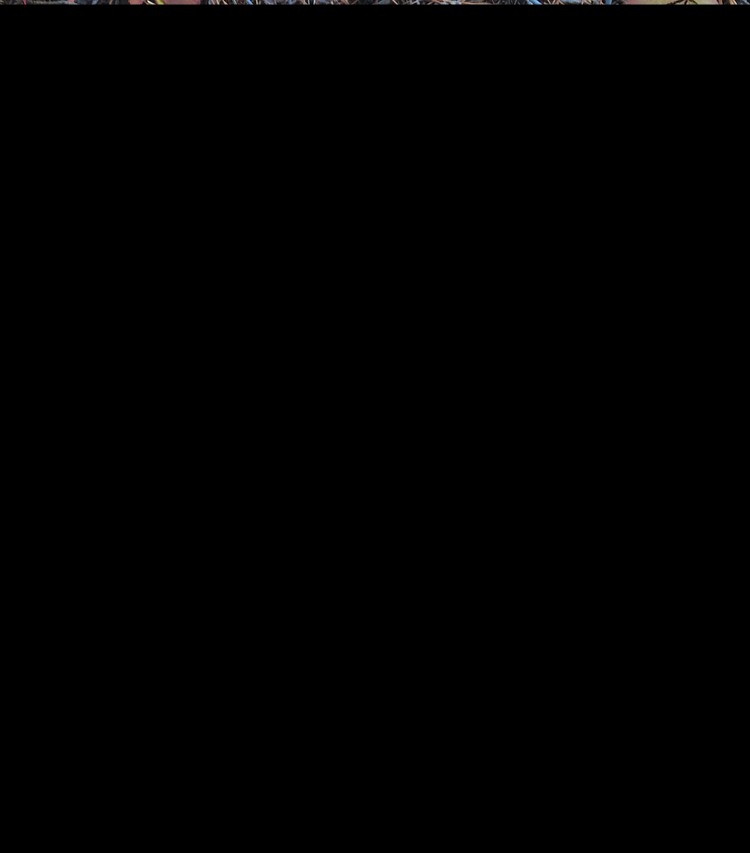
An *Amanita muscaria* var. *guessowii* mushroom collected from the location where the patient reported collecting the mushrooms that resulted in intoxication following consumption — Minnesota, October 2018 Photo / Minnesota Mycological Society

*A. muscaria* mushrooms can contain ibotenic acid and muscimol, which are structurally similar to glutamate and gamma-Aminobutyric acid, respectively; ibotenic acid is capable of producing central nervous system excitation (e.g., hallucinations, agitation, or seizures), and muscimol causes central nervous system depression (*1*). Ingestion of *A. muscaria* can also cause gastrointestinal symptoms (*2*). An Internet search revealed multiple recipes by avid foragers for *A. muscaria* reported as safe for consumption; however, *A. muscaria* toxins might not be deactivated by cooking (*3*).

In 2006, an outbreak occurred among two Hmong^†^ families in Minnesota who consumed *Amanita bisporigera*; this mushroom produces amatoxin, which is associated with gastrointestinal distress, liver failure, and high mortality. The outbreak affected nine persons and resulted in one death. Outreach efforts were focused on Hmong residents, including new arrivals in Minnesota (*4*).

Although the *A. muscaria* mushroom consumed by the patient in this report did not contain amatoxin, the event underscored that mushroom intoxications continue to be a concern for newly arrived persons accustomed to foraging in their home countries, who might not be familiar with local mushroom ecology. Because some local toxic mushrooms might resemble edible mushrooms found in Southeast Asia, MDH reached out to the Karen Organization of Minnesota and sought to identify additional mushroom intoxication cases, increase awareness among community leaders, and initiate community messaging about potential dangers of wild mushroom foraging. No additional mushroom intoxication cases were identified. Newly arrived persons might benefit from education concerning dangers associated with and the importance of avoiding consumption of wild mushrooms.

## Footnotes

* Karen are one of nine ethics groups in Burma. Approximately 17,000 Karen live in Minnesota, the largest Karen community in the United States.

† An ethnic group with roots in Southeast Asia. Hmong migrants began arriving in Minnesota in 1975 as refugees from the Vietnam War; approximately 66,000 Hmong live in Minnesota.

## References

[1] Michelot D, Melendez-Howell LM. *Amanita muscaria*: chemistry, biology, toxicology, and ethnomycology. Mycol Res 2003;107:131–46. 10.1017/S0953756203007305 12747324

[2] Moss MJ, Hendrickson RG. Toxicity of muscimol and ibotenic acid containing mushrooms reported to a regional poison control center from 2002–2016. Clin Toxicol (Phila) 2019;57:99–103. 10.1080/15563650.2018.1497169 30073844

[3] Tsunoda K, Inoue N, Aoyagi Y, Sugahara T. Change in ibotenic acid and muscimol contents in *Amanita muscaria* during drying, storing or cooking. Shokuhin Eiseigaku Zasshi 1993;34:153–60. 10.3358/shokueishi.34.153

[4] Holzbauer SM, Anderson D, Gerenday A, Outbreak of mushroom poisoning caused by *Amanita bisporigera*, the eastern American destroying angel—Minnesota, 2006. Proceedings of the 56th Annual Epidemic Intelligence Service Conference; April 16–20, 2007; Atlanta, GA. https://www.cdc.gov/eis/downloads/2007.EIS.Conference.pdf

